# The advances of DEAD-box RNA helicase 17 in chronic non-infectious diseases

**DOI:** 10.3389/fcvm.2025.1691840

**Published:** 2025-11-14

**Authors:** Mi Xiong, Qiang Wang, Ting Wang, Meiling Li, Xiangxiang Deng, Jia Wang, De Li, Yongjian Yang, Xiongshan Sun

**Affiliations:** Department of Cardiology, The General Hospital of Western Theater Command, Chengdu, Sichuan, China

**Keywords:** chronic non-infectious diseases, DDX17, structure, function, tumors, cardiovascular diseases

## Abstract

DEAD-box RNA helicase 17 (DDX17), a key member of the DEAD-box family, is vital in cellular physiological processes. This review summarizes its structural properties, distribution, functions, disease associations, and research trends. Structurally, DDX17 has a conserved DEAD-box domain with RNA-dependent ATPase and helicase activities, producing p72 and p82 isoforms. It distributes in the nucleus and cytoplasm, highly expressed in cardiomyocytes and neuronal tissues. Functionally, DDX17 regulates RNA metabolism, DNA repair, and protein interactions. It is linked to chronic non-infectious diseases: promoting tumor progression via pathways like Wnt/β-catenin; protecting myocardial function in cardiovascular diseases; and involving in neurological disorders.This review provides insights for exploring its biological functions and clinical applications.

## 1 Introduction

The DEAD-box is a type of RNA helicase widely present in eukaryotes, deeply involved in multiple key steps of RNA metabolism, including transcription, splicing, translation and RNA degradation (1). DDX17, with its unique structure and functions, plays a pivotal role in gene expression regulation and cell signal transduction (2).

In the regulation of gene expression, DDX17 can interact with estrogen receptors, androgen receptors, etc. as a transcriptional co-activator, and also regulate the chromatin environment to affect gene transcription by binding to long non-coding RNA (lncRNA) such as steroid receptor RNA activators (SRA) (3). At the RNA processing level, its regulation of precursor mRNA splicing, microRNA (miRNA) maturation and ribosomal RNA processing directly shapes the dynamic balance of the intracellular transcriptome (4, 5). It is worth noting that recent studies have revealed a close association between DDX17 and chronic non-infectious diseases. In tumors, DDX17 promotes cell proliferation by activating pathways such as Wnt/*β*-catenin and MYC (6). In cardiovascular diseases, DDX17 protects myocardial function by maintaining mitochondrial homeostasis (7, 8). In neurological diseases such as amyotrophic lateral sclerosis, the interaction between DDX17 and mutant FUS proteins is involved in neuronal protection (9, 10). The current review will systematically sort out the structure, function and disease association of DDX17, providing new ideas for the research of chronic non-infectious diseases (NCDs).

## 2 Basic characteristics of DDX17

### 2.1 Basic structure

DDX17 is a highly conserved RNA helicase containing a DEAD-box domain with RNA-dependent ATPase and RNA helicase activities (11). After silence of DDX17, the levels of the extended transcripts increased by 2–10 times. These elongated mRNAs are due to attenuated RNA cleavage or altered transcriptional termination. Knocking down DDX17 impairs the 3'-end processing of *de novo* transcripts and the transcriptional termination of a large number of genes. Furthermore, DDX17 splicing variability is evident after silencing, affecting nearly 20% of the expressed genes. 34% of genes exhibiting defective polyadenylation site (PAS) utilization are also affected at the splicing level, suggesting that transcriptional read-through may be associated with global deregulation of co-transcriptional RNA processing for this subset of genes (12). DDX17 also plays a role in the MUS81-LIG4-ELL pathway: By resolving the R-loop at the replication-repair conflict (TRC) site, it alleviates the topological barrier of the replication fork, enabling MUS81 to mediate the cleavage of the replication fork and the subsequent connection process, ultimately achieving replication restart (13). At the same time, studies have shown that DDX17 may first partially unwind the short-chain RNA-DNA heteroduplex, generating a 5' RNA protrusion end, and then recruit SETX (a progressive helicase) to complete the unwinding of the long R-loop, thereby ensuring efficient R-loop unwinding and the smooth restart of the replication fork (13, 14).

### 2.2 Unique structure

The mRNA of DDX17 has a unique structure, which contains a long noncoding region with an open reading frame starting at a specific position, and can be translated by non-AUG start codons to produce p72 and p82 isoforms with different N-terminal sequences (15). p72 is the main isoform of DDX17, which has a molecular weight of 72 kDa, and contains an intact DEAD-box structural domain and other structural domains. p82 is another isoform of DDX17 with a molecular weight of 82 kDa. Compared with p72, p82 contains an additional sequence at the N-terminus that may be involved in nucleoplasmic shuttling and functional regulation of DDX17 (15). For instance, p82 may play a role in nucleoplasmic shuttling (signaling sequences NLS and NES) and related cellular function regulation through its special structure in non-small cell lung cancer cells (16).

### 2.3 Distribution

DDX17 is predominantly distributed in the nucleus and cytoplasm of cells, and is found in a wide range of organs and tissues, including the heart, skeletal muscle, liver, kidney, and is particularly highly expressed in in cardiomyocytes, where it is involved in the growth, differentiation, and regulation of apoptosis, as well as in the maintenance of mitochondrial homeostasis (16–18). The expression of DDX17 in neuronal tissues is critical in the process of neuronal differentiation. DDX17 promotes miR-26a/b generation by ensuring the correct processing of CTDSP2/pri-miR-26a2 transcripts (19). DDX17 interacts in close proximity with the repressor element 1-silencing transcription factor (REST) in the nucleus and co-recruits to the promoters of REST-targeted genes, where it represses neuronal gene expression together with REST co-repressors such as EHMT2 (19).

## 3 The molecular mechanism of DDX17

### 3.1 RNA regulation

DDX17 is a multifunctional DEAD-box ATPase that is essential for RNA function and plays a key role in the maturation of microRNAs (miRNAs). Its core catalytic domain recognizes specific RNA sequences, such as the “RCAYCH” sequence through the RMFQ groove, which binds and remodels the 3' flanking regions of pri-miRs, enhancing the processing of Drosha, a ribonuclease III family enzyme (4). DDX17 not only affects the abundance of miRNAs by acting post-transcriptionally on the unloaded Ago2 proteins and thus participating in the regulation of Ago2 protein levels, but also interacts directly with Ago2 and indirectly regulates miRNA translational repression (20). However, the miRNA regulation mediated by DDX17 exhibits significant cell type specificity. This specificity is determined by cell type-specific cofactors, DDX17 itself, and the unique expression patterns of its downstream targets, as well as the cell-specific RNA regulatory networks (21, 22). Zhang et al. found that in glioma cell lines, DDX17 promotes the generation of miR-34-5p and miR-5195-3p by exerting its classical microprocessor complex function—that is, recognizing pri-miR sequences and assisting Drosha processing (21). These two miRNAs then directly target the 3' untranslated region of Beclin1, inhibiting its expression, and ultimately enhancing the migration, invasion ability, and malignant progression of glioma cells (21). Zhao et al. observed a non-classical regulatory pattern in colorectal cancer cell lines (22). DDX17 drives epithelial-mesenchymal transition and metastasis by down-regulating miR-149-3p, but this process does not rely on its microprocessor complex function (22). The study shows that DDX17 neither directly binds to pri-miR-149 nor alters the expression levels of pri-miR-149 or pre-miR-149, indicating that its regulatory effect may depend on other cell type-specific cofactors or regulatory cascades rather than directly recognizing pri-miR. DDX17 can also interact with Long Non-coding RNA Steroid Receptor RNA Activator and influence transcription by regulating the chromatin environment. DDX17 can enhance its interaction with the trithorax group complex and the polycomb repressive complex 2, thereby increasing the lysine 4 trimethylation level of histone H3 in specific genomic regions and promoting gene transcription (3). In terms of alternative splicing, DDX17 deletion can also affect the alternative splicing of the histone variant macroH2A1 pre-mRNA, resulting in an increase in the level of mH2A1.1 isoform and thereby altering the expression of genes related to redox metabolism (5).

### 3.2 DNA regulation

The DNA-RNA hybrid immunoprecipitation (DRIP) experiment showed that silencing of DDX17 significantly reduced DNA double-strand breaks (DSB)-induced DNA-RNA hybridization at all detection sites in the DIvA cell system (23). This indicates that DDX17 is essential for the formation of DNA-RNA hybrid structures after DSB formation. It is unclear whether DDX17 directly interacts with these known DNA repair genes. Since ubiquitination and signaling of histones in the DNA damage response (DDR) are dependent on DDX17, regulation of DNA-RNA hybridization by DDX17 may contribute to the remodeling of flanking chromatin in DSB (24, 25). Meanwhile, DDX17 can also interact with molecules such as RNF8, RNF168, *γ*-H2AX, and KU70, and jointly complete DNA repair (26).

### 3.3 Protein interaction

**Figure 1:**
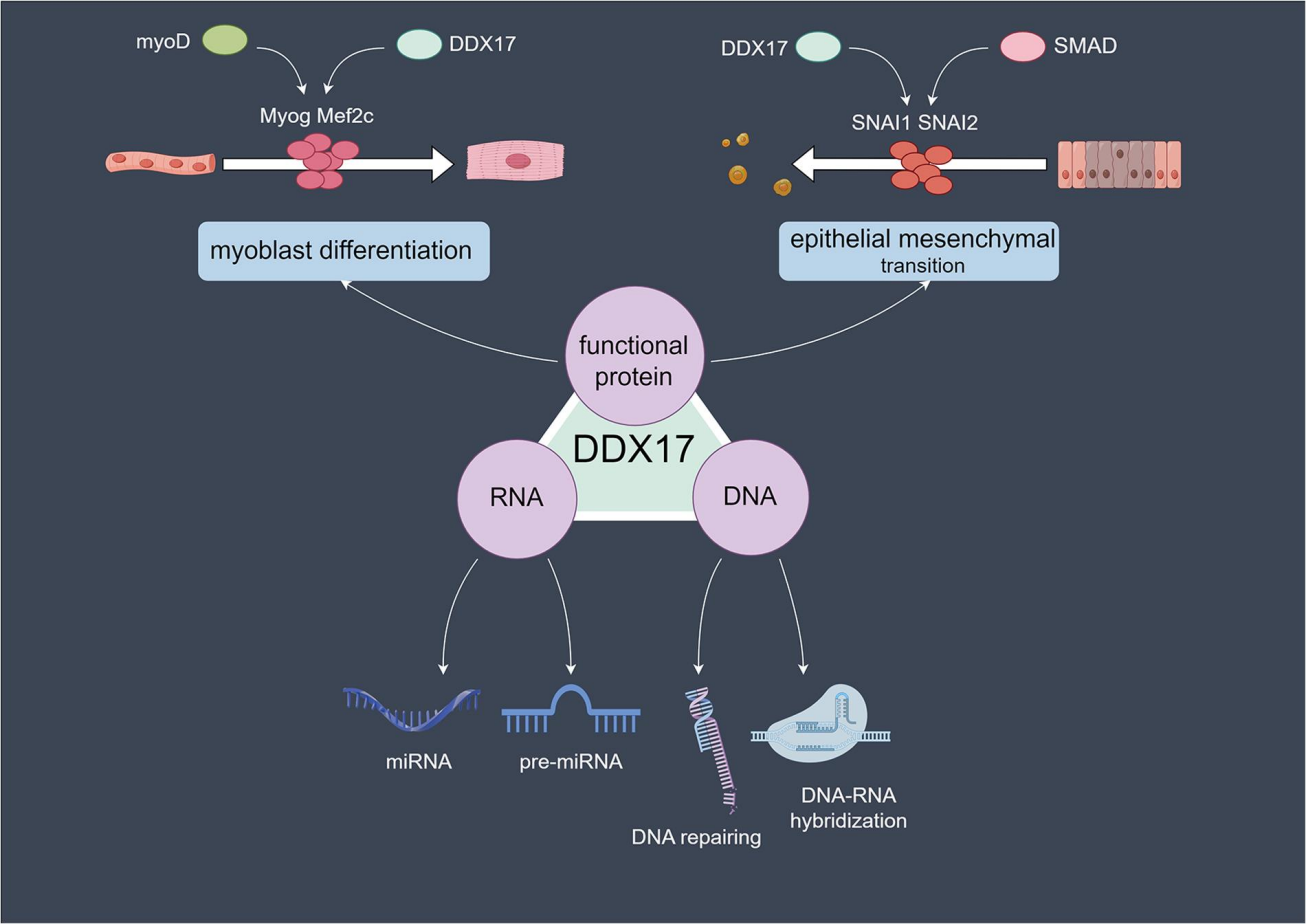
Molecular mechanisms of DDX17 in cellular processes: RNA, DNA, and protein-related functions.DDX17 acts on RNA (processing microRNAs and precursor microRNAs), DNA (repairing DNA and mediating DNA-RNA hybridization), and in protein-related cellular events, such as myoblast differentiation and epithelial-mesenchymal transition.in myoblast differentiation, DDX17 collaborates with myoD, influencing Myog and Mef2c, while in epithelial mesenchymal transition, it partners with SMAD to regulate SNAI1 and SNAI2.

DDX17 can function as a co-regulator of transcription factors such as MyoD and SMAD. Caretti et al. found that DDX17 can directly bind to Brg-1, TBP, and Pol II, suggesting that it promotes the binding of MyoD to key transcription initiation factors through a “molecular bridging” effect (11). At the same time, it assists in SWI/SNF-mediated chromatin remodeling, ultimately promoting the transcription initiation of myogenic genes such as Myog and Mef2c, ensuring the myogenic differentiation process (11). Moreover, this function of DDX17 does not rely on its RNA helicase activity, indicating that its role as a “scaffolding protein” is more crucial (11, 27). During TGF-*β*-induced epithelial-mesenchymal transition, DDX17 binds to SMAD transcription factors, regulates the expression of key epithelial-mesenchymal transition (EMT) transcription factors such as SNAI1 and SNAI2, and participates in the regulation of the transcriptional program, which promotes the cellular transition to the transcriptional state associated with EMT (Figure 1) (11, 28).

## 4 The regulatory roles of DDX17 in chronic non-infectious diseases

### 4.1 Tumors

**Figure 2:**
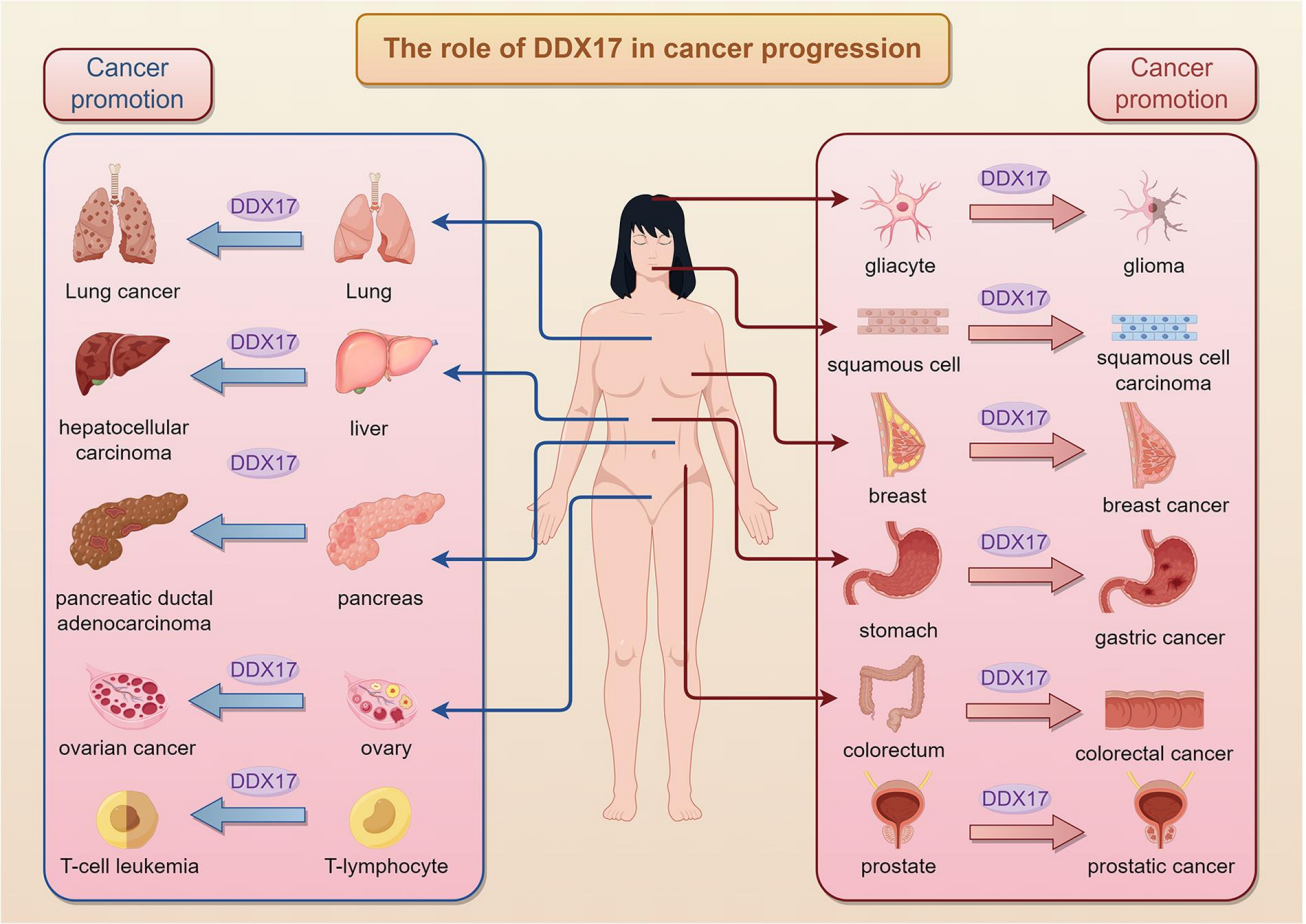
Role of DDX17 in cancer progression across human tissues.DDX17 is altered in the progression of multiple types of cancers and plays a key role in the process of tumorigenesis.It contributes to glioma from gliacytes, squamous cell carcinoma and breast cancer, highlighting its broad impact on carcinogenesis.

The expression level of DDX17 is significantly higher in cancers than that in normal tissues, suggesting that DDX17 may play a promotional or predictive role in tumorigenesis and development. In a variety of cancers, such as colon, breast, prostate, non-small cell lung, glioma, and hepatocellular carcinoma, DDX17 promotes cancer cell proliferation and inhibits apoptosis through the activation or inhibition of specific signaling pathways, thereby promoting tumorigenesis and progression (Figure 2) (21, 22, 29, 30). Rocaglates are a class of compounds, initially thought to be eIF4A antagonist, that exhibit potential in cancer therapy and protect against multiple infections, inducing GEF-H1 protein expression and JNK phosphorylation in tumor cells, triggering tumor cell death without affecting healthy organs (31). DDX17 plays an important role in the cellular response to Rocaglates-type compounds. Its translational activity is enhanced after Rocaglates treatment and participates in the remodeling of the translation machinery (32). Moreover, the expression change of DDX17 affects a series of cellular processes. Silencing of DDX17 weakens JNK phosphorylation and the induction of GEF-H1, CD98hc, eIF4A2 and other proteins, suggesting that it plays a key role in the signaling pathways induced by Rocaglates (32). DDX17 may affect cell survival and apoptosis by regulating protein synthesis, thereby playing an important role in the molecular mechanism of drugs related to cancer treatment and the study of chemotherapy resistance.

#### 4.1.1 Liver cancer

In hepatocellular carcinoma cells (HCCs), DDX17 affects the expression of target genes such as E-cadherin and matrix metalloproteinase-2 (MMP-2) by binding and inhibiting the transcriptional activity of Klf4, which in turn promotes tumor cell migration, invasion, and proliferation, and correlates with a poor prognosis (33). DDX17 can induce intron 3 retention of PXN-AS1 in hepatocellular carcinoma cells to produce PXN-AS1-IR3, which activates the MYC signaling pathway and promotes tumor progression by binding to Tex10. DDX17 also acts as a co-regulator of transcription factors such as estrogen receptor alpha (ER*α*) to promote hepatocellular carcinoma progression (33, 34). Furthermore, the levels of DDX17 in serum and cancer tissues are also correlated with tumor metastasis. Fan Feng et al.(2024) found that lactylation of the K230/322 site of aldolase A (ALDOA) in hepatocellular carcinoma stem cells (LCSCs) attenuates its binding to DDX17, which promotes the entry of DDX17 into the nucleus, enhances the stemness characteristics of LCSCs, and then exacerbates the development of hepatocellular carcinoma. This research suggests that DDX17 has a critical role in the maintenance of stemness in liver cancer stem cells, and its function is regulated by ALDOA lactylation (35). Circular RNA circDDX17 produced by reverse splicing of exons 2–5 of the DDX17 gene is down-regulated in HCCs and liver cancer tissues. External expression of circDDX17 enhances sorafenib sensitivity, promotes apoptosis, and inhibits the EMT process, and also inhibits the activity of miR-21-5p by competitively binding to the miR-21-5p, thereby regulating the PTEN/PI3 K/AKT pathway and affecting HCC progression (36). The above studies suggest that DDX17 plays an important role in promoting hepatocellular carcinoma progression and predicting poor prognosis.

#### 4.1.2 Prostate cancer

SNHG20 is a class of regulatory non-coding RNAs that are more than 200 nucleotides in length, and are highly expressed in a wide range of cancers, such as colorectal, hepatocellular, gastric, osteosarcoma, cervical and non-small cell lung cancers, and is associated with poor patient prognosis (37–42). Xing-Cheng Wu et al. demonstrated in their research that SNHG20 acts as a ceRNA, by sponging and adsorbing the 26 microRNAs shared by DDX17, relieving the inhibition of these microRNAs on DDX17, and upregulating the mRNA and protein levels of DDX17. Functional experiments showed that knocking down DDX17 can completely reverse the carcinogenic phenotype induced by overexpression of SNHG20, and high expression of SNHG20 is significantly associated with high Gleason score (>7) and advanced tumor stage (≥T2c) in prostate cancer, suggesting that the function of DDX17 regulated by SNHG20 plays a role in carcinogenesis (43).

#### 4.1.3 Lung cancer

E-cadherin is regarded as a tumor suppressor and plays an important role in the development of various cancers. In non-small cell lung cancer (NSCLC), DDX17 dissociates the complex formed by E-cadherin and *β*-catenin, promotes nuclear translocation of *β*-catenin and enhances its transcriptional activity, thereby activating the Wnt/*β*-catenin pathway and enhancing cellular resistance to gefitinib (16). This provides a potential therapeutic target for overcoming gefitinib resistance in NSCLC patients. Liu et al. (2022) found that DDX17 can also affect lung cancer development by regulating MYL9 and MAGEA6 (6). *MYL9* is the gene encoding myosin light chain 9, which is regulated by DDX17 in lung cancer and mediates the regulation of actin cytoskeletal rearrangement and cellular adhesion. Overexpression of MYL9 restores the adhesion ability of DDX17-deficient cells and also promotes stress fiber and adhesion spot formation. *MAGEA6* is the gene encoding melanoma-associated anti-6, belongs to the MAGEA gene family, and is positively regulated by DDX17. MAGEA6 inhibits cellular autophagy through the ubiquitination degradation of AMPK*α*1 by TRIM28 E3 ubiquitin ligase (6, 44, 45). DDX17 can bind the mRNAs of *MYL9* and *MAGEA6* to promote the expression of MYL9 and MAGEA6, regulate the process of cytoskeletal reorganization and autophagy in lung cancer cells, thus promoting the proliferation, migration, invasion and tumor growth of lung adenocarcinoma cells (6).

#### 4.1.4 Pancreatic cancer

In pancreatic cancer, DDX17 affects the malignant phenotype by regulating both caspase 9 and mH2A1 (46). Caspase 9 is a cysteine-aspartate protease that plays a central role in apoptosis. Variable splicing of the precursor mRNA of caspase 9 produces both pro-apoptotic Caspase 9a and anti-apoptotic Caspase 9b (47). mH2A1 is a histone variant whose variable splicing of the precursor mRNA produces both anti-invasive mH2A1.1 and pro-invasive mH2A1.2 isoforms (5). When inflammatory factors such as leukemia inhibitory factor and interleukin-6 inhibit the production of transfer RNA fragment-21, AKT1/2-mediated phosphorylation of heterogeneous nuclear ribonucleoprotein L (hnRNP L) is enhanced, which allows hnRNP L to interact with DDX17 to form the variable splicing complex. The complex causes variable splicing of caspase 9 and mH2A1 precursor mRNAs, leading to increased production of Caspase 9b and mH2A1.2, and thus enhances tumor metastasis (46–48). Parmanand Malvi et al. (2023) found that DDX17 is one of the phosphorylated target molecules of mitogen and stress-activated protein kinase 1 (MSK1), which exerts its pro-tumorigenic role in pancreatic ductal adenocarcinoma (PDAC). Knockdown of DDX17 results in reduced colony formation, decreased invasive ability, and increased apoptosis in PDAC cells. Whereas, overexpression of DDX17 partially restored these phenotypic alterations caused by MSK1 deletion (49).

#### 4.1.5 Stomach cancer

DDX17 is up-regulated in gastric cancer cells with acquired resistance to the combination of cisplatin and fluorouracil (CF) chemotherapy, and is identified as a pivotal gene in the protein-protein interaction network, which is closely related to the overall survival of patients (50). The poor overall survival of gastric cancer patients with high expression of DDX17 suggests that DDX17 may play an important role in the mechanism of drug resistance in gastric cancer. Therefore, DDX17 may be a potential target for the treatment of gastric cancer and overcoming chemotherapy resistance (51).

#### 4.1.6 Ovarian cancer

The expression levels of long non-coding RNA FAM225B and protein disulfide isomerase family member 4 (PDIA4) are reduced in ovarian cancer cells (52). FAM225B overexpression inhibits ovarian cancer cell proliferation, migration, invasion, while promotes apoptosis. PDIA4 overexpression also inhibits tumor cell progression (53). DDX17 not only directly binds to FAM225B, but also binds to the PDIA4 promoter to promote the expression of PDIA4. In ovarian cancer cells, the binding of FAM225B to DDX17 upregulates the expression of PDIA4, which in turn inhibits the progression of ovarian cancer cells (53, 54). This suggests that DDX17 promotes the proliferation, migration and invasion of ovarian cancer cells, providing a new potential target and theoretical basis for ovarian cancer research.

#### 4.1.7 Breast cancer

Silencing of DDX17 expression significantly inhibits the expression of estrogen-dependent genes (e.g., *pS2*, *Cathepsin D*) and the estrogen-dependent growth of MCF-7 and ZR75-1 breast cancer cells. Immunohistochemical analyses of 233 patients with ER*α*-positive breast cancers suggest that higher DDX17 expression is associated with better prognosis and is positively associated with HER2 negativity and progesterone receptor (55). Moreover, in AIB-1 positive tumors, the presence of DDX17 is associated with lower HER2 positivity, suggesting the involvement of DDX17 in ER*α*-mediated suppression of HER2 expression (55, 56). DDX17 interacts with SOX2, promotes binding of SOX2 to target genes, affects downstream gene expression, and enhances the tumorigenic and stem cell-like features (57).

#### 4.1.8 Glioma

In gliomas, DDX17 expression is significantly elevated, which inhibits the expression of autophagy-associated protein Beclin1 by enhancing the biosynthesis of miR-34-5p and miR-5195- 3p, thus promoting the processes of malignant migration, invasion, and apoptosis of glioma cells (21). Meanwhile, DDX17 forms a transcriptional complex with Jagged1 protein-derived JICD1 and SMAD3, TGIF2. This complex transcriptionally activates EMT-related genes such as TWIST1 by binding to specific elements, thus promoting the migration and invasion of glioma cells, a process independent of the TGF-*β* signaling pathway (58). In addition, analysis of enhancer RNA (eRNA)-regulated immune-related genes (IRGs) indicates that DDX17 is one of the key genes regulated by eRNAs, which may affect glioma development by influencing the infiltration pattern of immune cells and altering the tumor microenvironment (59). Several studies have demonstrated the importance of DDX17 in glioma development. The aberrant expression of DDX17 is closely related to the clinicopathological features of glial tumors, providing important clues for an in-depth understanding of the pathogenesis of gliomas and the development of new therapeutic strategies.

#### 4.1.9 Squamous cell carcinoma

In studies related to oral tongue squamous cell carcinoma (OTSCC) and buccal squamous cell carcinoma (BSCC), DDX17 serves as a pivotal gene in the PPI network constructed on the basis of the target genes of hsa-miR-136 and hsa-miR-377 and shows a significant coregulatory role in this network (60). In addition, high expression of DDX17 in head and neck squamous cell carcinoma (HNSCC) is associated with better prognosis, which implies that DDX17 may participate in certain biological process together with these genes during the development of squamous cell carcinoma and inhibit tumor development (60).

#### 4.1.10 Colorectal cancer

In colorectal cancer, DDX17 is highly expressed and associated with poor prognosis. The mechanism is mainly through the miR-149-3p/CYBRD1 pathway. Upregulation of DDX17 promotes the expression of mesenchymal markers and inhibits the expression of epithelial markers, which contributes to EMT and enhances metastatic ability of cancer cells (22). RNA sequencing analysis has revealed that DDX17 negatively regulates miR-149-3p expression, which suppresses the migration and invasion of colorectal cancer cells (61). Further studies found that CYBRD1 is a direct target gene of miR-149-3p. DDX17 reduces the 3’-UTR binding of miR-149-3p to CYBRD1 by down-regulating miR-149-3p, which in turn increases CYBRD1 expression and intracellular iron levels, and ultimately promotes EMT process and metastasis of colorectal cancer cells (22, 62).

#### 4.1.11 Leukemia

In adult T-cell leukemia, DDX17 is an important molecule interacting with the HBZ protein of human T-cell leukemia virus type 1 (HTLV-1), which interacts and partially co-localizes as a member of the family of RNA splicing-associated proteins in the interactions group of HBZ in the nuclei of leukemia cells (63). RNA sequencing analysis showed that HBZ can alter the transcription of many genes and affect different splicing events. Some of the exons regulated by HBZ are also target exons of DDX17, suggesting that DDX17 may be involved in HBZ-mediated alterations of the host transcriptome, and thus potentially plays a role in HTLV-1-associated leukemogenesis (63, 64).

### 4.2 Cardiovascular diseases

#### 4.2.1 Heart failure

DDX17 expression is downregulated in the mouse myocardium of heart failure and myocardial injury. Cardiomyocyte-specific knockdown of DDX17 promotes autophagic flux blockade and cardiomyocyte apoptosis, leading to progressive cardiac dysfunction, maladaptive remodeling, and progression to heart failure, whereas restoration of DDX17 expression protects the heart against pathological stresses (18). Further studies have shown that DDX17 binds to BCL6 and jointly participates in inhibiting the expression of the downstream gene *Drp1*, thereby preventing excessive mitochondrial fission in the myocardium and maintaining its stability and function (18). When cardiomyocytes are damaged, the expression of DDX17 decreases, and the transcriptional inhibitory function of BCL6 also decreases (18). This in turn leads to abnormal elevation of DRP1 expression, increased mitochondrial fission, disruption of normal mitochondrial homeostasis, and loss of myocardial cells and reduced cardiac function (18, 65). Testing of left ventricular myocardial biopsy samples from clinical heart failure patients also suggests a significant positive correlation between patients' left ventricular ejection fraction (EF%) and DDX17 expression in myocardial tissue (18, 66).

#### 4.2.2 Myocardial injury

DDX17 protects against doxorubicin (DOX)-induced cardiomyocyte injury by inhibiting estrogen receptor *α* (ER*α*) activation and reducing DOX-induced apoptosis (8). During myocardial growth, long-chain non-coding RNA CPhar plays an important role in myocardial physiological growth by interacting with DDX17 and recruiting DDX17 to segregate C/EBP*β*, which in turn regulates the downstream factor ATF7 (17). During myocardial ischemic/reperfusion injury, CPhar serves as a key regulator of exercise-induced cardio-protection, which triggers cardiac physiological hypertrophy and functional recovery by recruiting DDX17 to down-regulate ATF7. Meanwhile, DDX17 also plays a protective role in DOX-induced myocardial injury by activating the PI3K/Akt pathway (7). DDX17 expression is upregulated after growth hormone-releasing peptide (Ghrelin) treatment. Further research demonstrates that overexpression of DDX17 induces the expression of cardiomyocyte markers *α*-MHC and *β*-catenin. Additionally, Ghrelin can promote the differentiation of adipose tissue-derived mesenchymal stem cells (ADMSCs) to cardiomyocytes through DDX17-mediated regulation of the SFRP4/Wnt/*β*-catenin axis, which provides both a scientific basis for understanding the role of DDX17 in myocardial differentiation as well as in the clinical treatment of cardiac injury-related diseases (67).

#### 4.2.3 Aortic dissection

**Figure 3:**
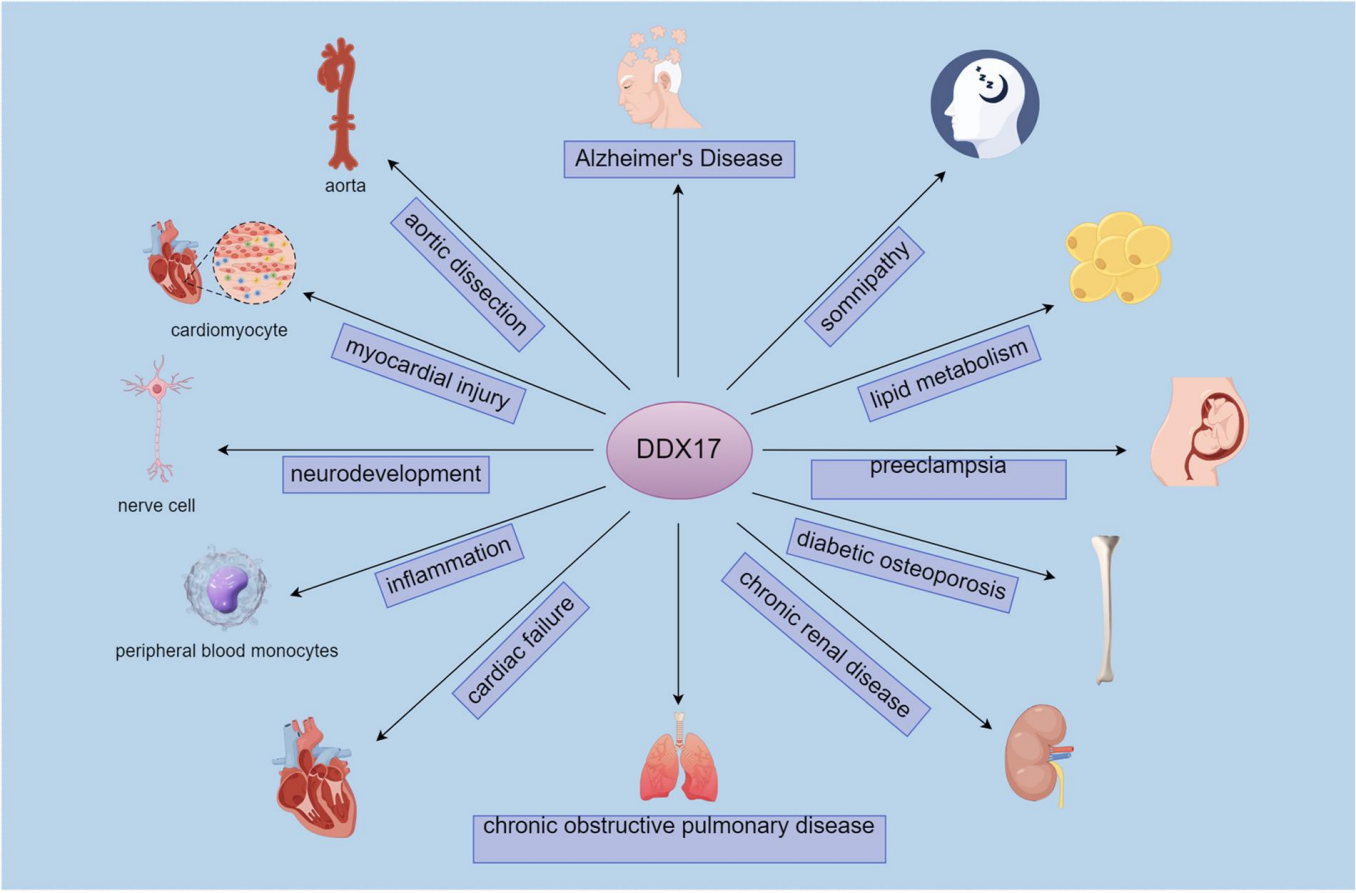
DDX17-Associated pathophysiological roles and target cells/tissues.DDX17 connects to conditions like Alzheimer's disease, aortic dissection, and chronic renal disease, alongside target cells/tissues such as cardiomyocytes, nerve cells, and the aorta.It links to neurological (Alzheimer's, neurodevelopment), cardiovascular (aortic dissection, myocardial injury), and metabolic/renal (lipid metabolism, chronic renal disease) pathways, via cells like nerve cells and peripheral blood monocytes.

DDX17 is identified as one of the key genes associated with m6A modification by comprehensive analysis of gene expression data and methylation data in aortic dissection (AD). In AD patients, DDX17 expression is reduced. As a member of the DEAD box family, DDX17 is associated with the regulation of vascular smooth muscle cells, possibly through synergistic action with DDX5 to regulate vascular smooth muscle cell growth and division (68). Immune infiltration analysis showed that DDX17 correlates with a variety of immune cells, such as activated NK cells, M2-type macrophages and resting mast cells. These studies suggest that DDX17 may be a potential target for AD diagnosis and treatment (69, 70). (Figure 3)

### 4.3 Diseases of the nervous system

Sarcomeric fusion protein (FUS) is an RNA-binding protein closely related to neurodegenerative diseases such as amyotrophic lateral sclerosis (ALS). DDX17 has been found to be closely associated with FUS in ALS (9, 10). RNA sequencing and comparative analysis of wild-type and FUS-mutant Drosophila brains shows that DDX17 is one of the targets significantly down-regulated by mutant FUS. *In vitro*, mutant FUS induces nuclear DDX17 translocated to cytoplasmic stress granules. FUS also interacts with DDX17 through the RGG1 structural domain of FUS. Overexpression of DDX17 reduces the aggregation and retention of mutant FUS in cytoplasmic stress granules and increases its solubility. In induced pluripotent stem cells with mutant FUS, DDX17 upregulation can repair the damaged DNA and reduce neuronal apoptosis. Rm62, a homologous gene of DDX17, modifies the toxicity of FUS in Drosophila. Upregulation of DDX17 improves the locomotor ability and survival of FUS-expressing Drosophila (71). These studies indicate that DDX17 plays an important role in FUS-associated ALS, which provides new perspectives for an in-depth understanding of the pathogenesis and therapeutic strategies of ALS.

*de novo* variation in a single allele of *Ddx17* can lead to neurodevelopmental disorders (72). 11 patients display intellectual disability, language and motor delays. Functional experiments reveal that DDX17 plays an important role in neuro-development. Knockdown of DDX17 affects neuronal migration and axonal development, leading to reduced axonal growth in mouse and African clawed toad models. The heterozygous tadpoles also suffer from working memory deficits. Transcriptome analysis shows that DDX17 is involved in regulating several genes related to the development of the nervous system (72).

In Alzheimer's disease (AD), DDX17 expression is up-regulated. Overexpression of DDX17 enhances BACE1 (*β*-secretase 1) translation, which in turn promotes amyloidogenesis (73, 74). DDX17 promotes BACE1 translation in a 5′ UTR-dependent manner by interacting with the 5′ UTR of *Bace1* mRNA, thus increasing BACE1 expression and A*β* (β-amyloid) production without affecting ADAM10 (75, 76). Whereas, translational inhibitors blocked the regulation of BACE1 by DDX17, further suggesting that DDX17 acts through a translational mechanism to regulate BACE1. These studies provide a novel perspective for understanding the pathogenesis of AD and searching potential therapeutic targets (77).

### 4.4 Chronic obstructive pulmonary disease (COPD)

Bioinformatics analysis identified DDX17 as a possible candidate gene associated with T cell activation and differentiation in lung tissues of COPD model (78). Furthermore, in the lung tissues of the mouse emphysema model, the mRNA and protein expression levels of DDX17 are significantly elevated, suggesting that DDX17 may be involved in the pathogenesis of COPD, but the detailed roles and mechanisms are not clear (78). These studies offer theoretic basis for subsequent study exploring the function of DDX17 in COPD (78).

### 4.5 Chronic kidney disease (CKD)

DDX17 expression is decreased in peripheral blood mononuclear cells of CKD patients as analyzed by weighted gene co-expression network analysis (WGCNA) (79). DDX17 plays an important role in innate immune defense against viral invasion. In CKD model, lower expression of DDX17 may be associated with immunodeficiency, which in turn causes the progression of renal disease. Therefore, DDX17 is expected to be a potential biomarker or a therapeutic target for CKD (79–81).

### 4.6 Pre-eclampsia (PE)

WGCNA analysis showed that DDX17 is one of the key genes associated with PE, with a high node degree in the PPI network (82). This study also identified multiple genes which have high correlation with DDX17 with high node degree, suggesting that DDX17 may synergize with other genes to influence the developmental process of PE. This synergy of DDX17 with other genes is more likely to promote the onset and progression of PE (82). In addition, DDX17 also plays an important role in RNA-associated functions in the PPI network, which may be involved in the pathogenesis of PE through influencing RNA metabolism and other associated pathways.

### 4.7 Diabetic osteoporosis (DOP)

The expression of DDX17 is decreased in high glucose-treated bone marrow mesenchymal stem cells and participates in the regulation of the osteogenic differentiation process of BMSCs as a target gene of miR-9-5p (83). The expression alteration of DDX17 *in vivo* is also correlated with different stages of DOP. Therefore, DDX17 is also expected to be a potential target for the diagnosis or treatment of DOP.

### 4.8 Sleep disorders

Identified by machine learning algorithms, DDX17 is one of the pivotal genes that is closely associated with sleep disorders, and is involved in a variety of cellular processes associated with RNA secondary structure alterations, and plays a core role in estrogen and testosterone signaling pathways. DDX17 influences the mRNA processing of hormones and neurotransmitters involved in sleep regulation, which in turn regulates sleep cycle and quality (84–86). This helps to explain how DDX17 may influence sleep disorders.

### 4.9 Inflammation

Activation of inflammasome is critical in host defense against pathogens. DDX17 is a sensor of endogenous short-intercalated nuclear elements (SINE RNAs), and interacts with SINE RNAs to promote the assembly of NLRC4, NLRP3, and apoptosis-associated speck-like protein containing a CARD (ASC) into a complex. The assembled NLRP3-ASC complex recruits and activates CASP1 and induces cytokines release (87, 88). In peripheral blood mononuclear cells from patients with systemic lupus erythematosus, DDX17-induced NLRC4 inflammasome is activated. Inhibition of DDX17-mediated NLRC4 inflammasome activation reduces the release of interleukin-18 (87, 89). In an animal model of age-related macular degeneration, inhibition of DDX17-mediated NLRC4 inflammasome activation prevents retinal pigment epithelial cell degeneration (87). These evidence strongly suggest that DDX17 plays a critical role in SINE RNA-drived sterile inflammatory diseases. Clinical studies have shown that DDX17 plays an important role in the progression of nonalcoholic steatohepatitis (NASH) as elevated expression of DDX17 is observed in the liver of NASH patients (90). DDX17 affects lipid metabolism and inflammatory response by synergistically interacting with CCCTC-binding factor and DDX5 and thus transcriptionally represses *Cyp2c29* gene expression. In addition, DDX17 is involved in the regulation of M1 macrophage activation and thus is associated with hepatic steatosis and fibrosis (90).

### 4.10 Lipid metabolism

In the oleic acid/palmitic acid (OA/PA)-induced lipid accumulation model in hepatocytes, DDX17 expression is significantly reduced. Overexpression of DDX17 markedly attenuated OA/PA-induced lipid accumulation in HepG2 and Hep1-6 cells, which is mainly attributed to the reduction of intracellular triglyceride content (91). Meanwhile,DDX17 overexpression significantly down-regulates the expression of genes associated with *de novo* fatty acid synthesis, suggesting that DDX17 may inhibit lipid synthesis and protect against lipid accumulation in hepatocytes treated by OA/PA, which provides a potential molecular target for the treatment of metabolism-associated fatty liver disease (91).

## 5 Conclusion and outlook

DDX17, as an important member of the DEAD-box RNA helicase family, exhibits multiple critical roles in cellular physiological processes and also disease development. The unique structural properties, such as the DEAD-box structural domain, RNA-dependent ATPase and RNA deconjugating enzyme activities, and the unique mRNA structure which can generate different isoforms, have endowed DDX17 with diverse functions. DDX17 is widely distributed in the nucleus and cytoplasm, and is involved in important physiological processes such as transcriptional regulation, RNA processing, and DNA repair. DDX17 acts synergistically with a variety of transcription factors and RNA-binding proteins, thus affecting gene expression, mRNA splicing and maturation, as well as protein function. DDX17 expression level varies abnormally in a variety of cancers. Therefore, DDX17 plays either an oncogenic or inhibitory role in tumor cell proliferation, migration, invasion, apoptosis, and drug resistance by interacting with different molecules, thus is involved in tumor progression or prediction. In cardiovascular system, DDX17 is closely related to the development of heart failure, repair of cardiac damage and myocardial differentiation. Additionally, DDX17 also plays an important role in various diseases involves other organs or pathological processes, including neurodegenerative diseases, inflammation-related diseases, chronic kidney disease, metabolism-related diseases.

Though DDX17 has been extensively and deeply investigated, there are still several aspects need to be further explored. Future studies about DDX17 may focus on more precise mechanism of DDX17 at the molecular level, such as searching for other proteins interacting with DDX17 and the other transcriptional or post-transcriptional modification. The specific or core node of DDX17 on the regulatory network in different diseases suggest that DDX17 may exert more extensive effects. Based on the core roles of DDX17 in RNA processing (such as pri-miRNA cleavage and mRNA splicing), protein interaction, and pathway regulation, its drug development can be targeted to design three types of strategies—small molecule inhibitors that specifically target the DEAD-box domain (to block the oncogenic pathways mediated by miRNAs in cancer) and peptide drugs that interfere with protein interactions (to stabilize the DDX17-BCL6 complex in heart failure or inhibit the DDX17-SMAD binding in cancer EMT). DDX17 has the potential to serve as a multi-disease clinical biomarker. The mRNA/protein levels of DDX17 in tissue samples are significantly correlated with the EF value of heart failure, the metastasis status of cancer, and the expression of cfRNA/exosomes in blood can be used as convenient detection indicators. RT-qPCR (with a detection limit of 10 copies/*μ*l) and IHC (specificity 82%, sensitivity 76% in colorectal cancer) are reliable methods, and they can also quantify the association between heart failure stage, cancer prognosis, and treatment response.
